# Use of the Nanofitin Alternative Scaffold as a GFP-Ready Fusion Tag

**DOI:** 10.1371/journal.pone.0142304

**Published:** 2015-11-05

**Authors:** Simon Huet, Harmony Gorre, Anaëlle Perrocheau, Justine Picot, Mathieu Cinier

**Affiliations:** Affilogic SAS, Nantes, France; Cardiff University, UNITED KINGDOM

## Abstract

With the continuous diversification of recombinant DNA technologies, the possibilities for new tailor-made protein engineering have extended on an on-going basis. Among these strategies, the use of the green fluorescent protein (GFP) as a fusion domain has been widely adopted for cellular imaging and protein localization. Following the lead of the direct head-to-tail fusion of GFP, we proposed to provide additional features to recombinant proteins by genetic fusion of artificially derived binders. Thus, we reported a GFP-ready fusion tag consisting of a small and robust fusion-friendly anti-GFP Nanofitin binding domain as a proof-of-concept. While limiting steric effects on the carrier, the GFP-ready tag allows the capture of GFP or its blue (BFP), cyan (CFP) and yellow (YFP) alternatives. Here, we described the generation of the GFP-ready tag from the selection of a Nanofitin variant binding to the GFP and its spectral variants with a nanomolar affinity, while displaying a remarkable folding stability, as demonstrated by its full resistance upon thermal sterilization process or the full chemical synthesis of Nanofitins. To illustrate the potential of the Nanofitin-based tag as a fusion partner, we compared the expression level in *Escherichia coli* and activity profile of recombinant human tumor necrosis factor alpha (TNFα) constructs, fused to a SUMO or GFP-ready tag. Very similar expression levels were found with the two fusion technologies. Both domains of the GFP-ready tagged TNFα were proved fully active in ELISA and interferometry binding assays, allowing the simultaneous capture by an anti-TNFα antibody and binding to the GFP, and its spectral mutants. The GFP-ready tag was also shown inert in a L929 cell based assay, demonstrating the potent TNFα mediated apoptosis induction by the GFP-ready tagged TNFα. Eventually, we proposed the GFP-ready tag as a versatile capture and labeling system in addition to expected applications of anti-GFP Nanofitins (as illustrated with previously described state-of-the-art anti-GFP binders applied to living cells and *in vitro* applications). Through a single fusion domain, the GFP-ready tagged proteins benefit from subsequent customization within a wide range of fluorescence spectra upon indirect binding of a chosen GFP variant.

## Introduction

Recombinant DNA technologies have continued to diversify since the early discovery of ligase [1,2] and restriction enzymes [3]; expanding the possibilities for gene manipulations and protein engineering. Restriction-free cloning [4] and Gibson DNA assembly [5] are two examples of the techniques now available, allowing respectively the insertion of a unique or multiple DNA fragments in a single reaction, which dramatically simplify the procedure for the construction of chimeric fusion proteins. This evolution of the DNA assembly techniques opens the way for the design of new tailor-made strategies to complement the already existing set of fusion partners and features.

Among the well-established fusion technologies, the green fluorescent protein (GFP), and its spectral variants, has been widely adopted for cellular imaging and protein localization. Its range of applications was broaden again by the development of an anti-GFP Nanobody companion tool (termed Nanotrap), a non-IgG binding protein able to induce the interference, mislocalization, or degradation of GFP fusion proteins *in vivo* [6,7]. More recently, anti-GFP DARPins have been developed and reported for similar protein interference study [8]. RFP-Nanotrap fusion has also been shown helping the visualization of protein–protein interactions in living cells [9]. Moreover, the genetic fusion of proteins to an anti-GFP binding domain has been described as an efficient strategy to promote *in vivo* dimerization [10] or stabilization of a whole complex [11]. These anti-GFP scaffolds, like most of the other artificially derived binding protein scaffolds (see [12] for a recent review of non-immunoglobulin scaffolds), share common features making them highly suitable as a fusion partner [13]: small size (< 20 kDa), cysteine-free, high solubility, good expression, and proper folding in various prokaryotic and eukaryotic host cells, without post-translation modification required for activity.

All these fusion-friendly features also readily apply to the Nanofitin scaffold. Nanofitins are derived from DNA binding Sac7d family and homologous OB-fold proteins, including recently described Sac7d [14–26] and Sso7d [24,27–34] engineered mutants. Discovered in extreme natural conditions (pH 2 and 85°C) from an archaebacterial protein, the Nanofitin scaffold extends the scope of the properties of its derived binders with high stability to wide ranges of pH, temperature and chemicals, while presenting high specificity and affinity (up to pM range [16]). A remarkable use of concomitant stability and binding activity of a fused OB-fold protein is known as the commercially available Phusion DNA polymerase, which consists in C-terminal fusion of Sso7d dsDNA binding domain to *Pfu* DNA polymerase [27].

In this study, we identified a Nanofitin with nM affinity towards protein members of the GFP family (Blue, Cyan, Green, and Yellow fluorescent proteins) that also keeps unique features of the Nanofitin scaffold regarding its simplicity, stability, expression, and solubility. We expect this anti-GFP Nanofitin to be suitable for *in vitro* to live cell applications, in a similar way to previously discovered anti-GFP binders (including outlined Nanobodies and DARPins, or more recently described αReps [35]). While most of the previous studies are application-oriented, we would rather focus on the use of an anti-GFP Nanofitin as a generic fusion tag, named GFP-ready tag, and propose an extension of the Nanofitin-based fusion technology applied to the expression of heterologous recombinant proteins. In this study, we demonstrated a proof-of-concept within the framework of a GFP-ready fusion to human TNFα. While remaining fully active, the GFP-ready tagged-TNFα gains the ability to bind spectral variants of GFP, offering a versatile system for its capture and detection with a customizable fluorescence spectrum.

## Materials and Methods

### *In vitro* generation of anti-GFP Nanofitins

#### Biotinylation of antigens

For the selection and the identification of the clones, biotinylated StrepTagII-GFP was utilized. The biotinylation was performed by incubation of a 110 μM solution of the target protein with a 5-fold molar excess of sulfosuccinimidyl-6-(biotinamido) hexanoate (Sulfo-NHS-LC-LC-Biotin, Pierce) in PBS (Sigma-Aldrich) on ice for 1 h. The biotinylated protein was buffer-exchanged using protein desalting spin columns (Pierce) equilibrated in 20 mM Tris–HCl, 150 mM NaCl pH 7.4 (TBS). The degree of biotinylation was determined, using a 4-Hydroxyazobenzene-2-carboxylic acid (HABA/Avidine) assay (Sigma-Aldrich), as being about 2 molecules of biotin per protein molecule.

#### Ribosome display selection rounds and isolation of clones

The combinatorial library of Nanofitins was prepared as previously described [16,21]. Briefly, the library was assembled by two successive overlapping PCR from degenerated oligonucleotides encoding NNS triplets (N = A, C, T or G and S = C or G). Eventually, a final PCR step added the 5’- and 3’-flanking region necessary for ribosome display [36]. The PCR-amplified library was transcribed and the selection was done at 4°C as described by Mouratou *et al*. [16,21]. Although adding the anti-ssrA oligonucleotide to the translation mix was described to improve the stability of the mRNA-ribosome-protein complex [37], no benefit was observed in our hand and the selection was performed in absence of anti-ssrA. Six rounds of selection were performed to isolate high-affinity binders. The pressure of selection was adjusted by gradually increasing the time in wash-steps during the 4 first rounds (8 washes of 30 s, 3 min and 15 min, respectively, for rounds 1, 2, and 3, then 4 washes of 15 min followed with 4 washes of 30 min for rounds 4 to 6) and then decreasing the quantity of the target protein used for the panning step for the two last rounds (15 pmol for rounds 1 to 4, 3.75 pmol for round 5 and 0.93 pmol for round 6).

Amplified DNA material from the sixth round was cloned between BamHI and HindIII restriction sites of the plasmid pQE-30 (Qiagen), and the ligation mixture was transformed into *E*. *coli* DH5α LacIq strains (Invitrogen). Clones selected on 2xYT medium plates containing 100 μg/ml ampicillin and 25 μg/ml kanamycin were inoculated into a deep-well plate containing 0.75 ml of 2xYT medium with 100 μg/ml ampicillin, 25 μg/ml kanamycin, and 1% glucose in each well. After overnight culture at 37°C while shaking at 600 rpm, 0.2 ml of each culture was used to inoculate another deep-well plate containing 1.25 ml of 2xYT medium supplemented with 100 μg/ml ampicillin, 25 μg/ml kanamycin, and 0.1% glucose per well. The plate was incubated at 37°C for 3 h while shaking at 600 rpm. Expression of the Nanofitin clones was induced by addition of 50 μl of Isopropyl β-D-1-thiogalactopyranoside at a final concentration of 0.5 mM and incubation at 30°C for 4 h with shaking at 600 rpm. Cells were pelleted by centrifugation (20 min at 2000*g*), and supernatants were discarded. Proteins were extracted with 100 μl of 1X BugBuster Protein Extraction Reagent (Novagen) per well with shaking for 1 h at room temperature, and 350 μl of TBS (20 mM Tris-HCl, 150 mM NaCl, pH 7.4) were added. Cell debris was pelleted by centrifugation (20 min at 2000*g*) and supernatants were used for screening purpose.

#### ELISA screening of the anti-GFP Nanofitins

Streptavidin (100 μl, 66 nM; Sigma-Aldrich) in TBS was immobilized in Maxisorp plate wells (Nunc) by overnight incubation at 4°C. Each of the following steps were run at room temperature, with shaking at 600 rpm for incubation steps. The wells were washed 3 times with 300 μl of TBS, then blocked with 300 μl of 0.5% BSA (bovine serum albumin; Sigma-Aldrich) in TBS for 1 h. The plate was flicked over and biotinylated StrepTagII-GFP (100 μl, 40 nM) in TBS with 0.5% BSA was allowed to bind for 1 h. Prior to each of the following incubation steps, the wells were washed 3 times with 300 μl of TBS containing 0.1% Tween 20. Crude *E*. *coli* extracts (100 μl, diluted 1:40 in TBS with 0.1% Tween 20) were applied to wells with and without immobilized antigen for 1 h. Revelation was then carried over by the addition of 100 μl of RGS His antibody HRP conjugate (Qiagen) diluted 1:4000 in TBS with 0.1% Tween 20 for 1 h, followed by the addition of 100 μl of *o*-Phenylenediamine dihydrochloride substrate (Sigma-Aldrich) solution at 1 mg/ml in revelation buffer (0.05 M citric acid, 0.05% hydrogen peroxide). Absorbance at 450 nm was measured using a Varioskan ELISA plate reader (Thermo Scientific).

### Construction of human tumor necrosis factor alpha fusions

Coding sequence of human tumor necrosis factor alpha (TNFα) was kindly provided by Prof. Sven Pfeifer (Martin Luther University of Halle-Wittenberg, Germany) as a fusion with a N-terminal Small Ubiquitin-like Modifier protein (SUMO) in a pET vector [38]. Gene coding for the chimeric construct SUMO-TNFα was sub-cloned in pQE30 vector by Gibson assembly [5], resulting in a StrepTagII SUMO-TNFα construct. The vector and SUMO-TNFα coding sequence were amplified by PCR using, respectively, the pairs of oligonucleotides Gpls01C_For (TAATGACTGAGCTTGGACTCC) and Gpls05N_Rev (GAACTGCGGGTGGCTCCAGCTTGCCATAGTTAATTTCTCCTCTTTAATGAATTC), or NStrep_SUMO_For (TGGAGCCACCCGCAGTTCGAAAAGGGATCCATGTCGGACTCAGAAGTCAATCAAG) and TNF_Stop_Rev (GAGTCCAAGCTCAGTCATTACAGCGCAATAATGCCAAAATAC). Linearized vector (100 ng) was mixed with 3 molar equivalents of the gene insert in a final volume of 5 μl. Then, 15 μl of the Gibson assembly mix (25% PEG-8000, 500 mM Tris-HCl, 50 mM MgCl2, 50 mM DTT, 1 mM Mix dNTPs, 5 mM NAD, 2U of T5 exonuclease, 12.5U of Phusion polymerase, 2000U of Taq ligase) were added and the solution was incubated for 1 h at 50°C. *E*. *coli* DH5α LacIq strains (Invitrogen) were transformed with 10 μl of the resulting material. Clones were selected on 2xYT medium plates containing 100 μg/ml ampicillin and 25 μg/ml kanamycin.

Construction of StrepTagII GFP-ready-TNFα followed a similar procedure but the GFP-ready-TNFα coding sequence was amplified as 2 separated fragments using, respectively for D8 and TNFα amplicons, the pairs of oligonucleotides Gpls05N_For (TGGAGCCACCCGCAGTTCGAAAAGGGATCCGTCAAGGTGAAATTC) and D8_GS_Rev (GCTACGCACCGAGCCCTTTTTCTCGCGTTCCGCA), or GS_TNF_For (GGCTCGGTGCGTAGCAGCAGCC) and TNF_Stop_Rev. Linearized vector amplified with oligonucleotides Gpls01C_For and Gpls05N_Rev was mixed with 3 molar equivalents of each fragment for the Gibson assembly reaction.

Construction of Histag SUMO-TNFα and Histag GFP-ready-TNFα expression vectors is described in “S1 Appendix”.

All the constructions were confirmed by Sanger sequencing (GATC biotech).

### Protein production and purification

GFP variants, Nanofitin mutants and human TNFα fusions were expressed in *E*. *coli* DH5α LacIq strains.

Briefly, precultures were grown overnight at 37°C in 2xYT medium with 1% glucose, 100 μg/ml ampicillin and 25 μg/ml kanamycin. Precultures were diluted 1:20 in 2xYT medium with 0.1% glucose, 100 μg/ml ampicillin and 25 μg/ml kanamycin, and grown at 37°C to mid-log phase (OD600 = 0.8–1.0). Then, protein expression was induced by addition of Isopropyl β-D-1-thiogalactopyranoside to the final concentration of 0.5 mM and the culture shaken at 30°C overnight. Bacteria were pelleted by 45 min centrifugation at 3220*g*. Cell pellets were resuspended in a pH 7.4 lysis buffer composed of 1X BugBuster Protein Extraction Reagent, 5 μg/ml DNaseI, 20 mM Tris, 500 mM NaCl, and 25 mM Imidazole. Cell lysis occurred at room temperature for 1 h and the suspension was centrifuged at 3220*g* for 45 min to remove cell debris. Histag-proteins were then purified from supernatants by immobilized metal ion affinity chromatography (IMAC), using His60 Nickel Superflow resin (Clontech) and a pH 7.4 elution buffer composed of 20 mM Tris, 500 mM NaCl, and 250 mM Imidazole.

For StrepTagII-proteins, lysis buffer was composed of 1X BugBuster Protein Extraction Reagent, 5 μg/ml DNaseI, 100 mM Tris and 150 mM NaCl, and the purification was performed by affinity chromatography, using Strep-Tactin Sepharose resin (IBA) and a pH 8.0 elution buffer composed of 100 mM Tris, 150 mM NaCl and 2.5 mM D-Desthiobiotin.

Additional endotoxin removal step was carried over for samples engaged in cell-based assay. First, samples were buffer-exchanged by dialysis against PBS (10 mM PO_4_^3-^, 2.7 mM KCl and 137 mM NaCl, pH 7.4; Sigma-Aldrich) or citrate buffer (100 mM citrate, 150 mM NaCl, pH 4.0), respectively, for D8-TNFα and SUMO-TNFα fusions. Then, samples were filtered on a Sartobind STIC PA anion exchanger (Sartorius). Finally, samples were dialyzed against PBS, filtered on Minisart hydrophilic membranes with 0.2 μm pore size (Sartorius), then stored in sterile conditions.

### Autoclaving resistance

Autoclaving of anti-GFP Nanofitins D8 was performed at 5 mg/ml in TBS, for 20 min at 121°C. Insoluble proteins in autoclaved and non-treated samples were pelleted by centrifugation during 5 min at 4°C and 20000*g*, and soluble proteins in supernatants were quantitated by absorbance measure at 280 nm. The residual binding to GFP was measured by ELISA, with StrepTagII-GFP (340 nM, 100 μl/well) in TBS immobilized on a Maxisorp plate (Nunc) by overnight incubation at 4°C. Each of the following steps were run at room temperature, while shaking at 600 rpm for incubation steps. The wells were washed 3 times with 300 μl of TBS, then blocked with 300 μl 0.5% BSA (Sigma-Aldrich) in TBS for 1 h. Prior to each of the following incubation steps, the wells were washed 3 times with TBS containing 0.1% Tween 20. Anti-GFP Nanofitins D8 (autoclaved or non-treated, 100 μl) were diluted to give the final concentration of 250 nM to 15 pM and were allowed to bind for 1 h in TBS with 0.1% Tween 20. Revelation was then carried over by the addition of 100 μl of RGS His antibody HRP conjugate (Qiagen) diluted 1:4000 in TBS with 0.1% Tween 20 for 1 h, followed by the addition of 100 μL of *o*-Phenylenediamine dihydrochloride substrate (Sigma-Aldrich) solution at 1 mg/ml in revelation buffer (0.05 M citric acid, 0.05% hydrogen peroxide). Absorbance at 450 nm was measured using a Varioskan ELISA plate reader (Thermo Scientific).

### Characterization of GFP-ready tagged TNFα

#### ELISA

Anti-TNFα monoclonal antibody (Infliximab) was kindly provided by Dr. Arnaud Bourreille (University Hospital of Nantes, France) and is also referred to as Remicade (Antibody Registry identifier: AB_2459635). Infliximab (10 μg/ml, 100 μl/well) in TBS was immobilized on a Maxisorp plate (Nunc) by overnight incubation at 4°C. Each of the following steps were run at room temperature, with shaking at 600 rpm for incubation steps. The wells were washed 3 times with 300 μl of TBS, then blocked with 300 μl 0.5% BSA (Sigma-Aldrich) in TBS for 1 h. Prior to each of the following incubation steps, the wells were washed 3 times with TBS containing 0.1% Tween 20. TNFα recombinant protein (StrepTagII-SUMO-TNFα or StreptagII-D8-TNFα, 100 μl, 250 nM), or empty buffer, was allowed to bind for 1 h in TBS with 0.1% Tween 20. Histag-GFP variants (100 μl, 250 nM) were then added to the wells and the plate was incubated for 1 h. Revelation was then carried over by the addition of 100 μl of RGS His antibody HRP conjugate (Qiagen) diluted 1:4000 in TBS with 0.1% Tween 20 for 1 h, followed by the addition of 100 μL of *o*-Phenylenediamine dihydrochloride substrate (Sigma-Aldrich) solution at 1 mg/ml in revelation buffer (0.05 M citric acid, 0.05% hydrogen peroxide). Absorbance at 450 nm was measured using a Varioskan ELISA plate reader (Thermo Scientific).

#### Binding kinetic assay

Binding kinetic parameters of the anti-GFP Nanofitin D8 for the GFP and color variants were measured by interferometry on Octet RED96 system (ForteBio). For purified D8 Nanofitin alone, the biotinylated protein was diluted to 0.5 μg/ml and directly loaded on streptavidin biosensors at 0.5 nm, then biosensors were allowed to equilibrate for 60 s. Binding kinetic was then evaluated by exposing simultaneously biosensors to various concentrations (100, 50, 25, 12.5, 6.25, 3.125, 1.256 and 0 nM) of Histag-GFP or spectral variants.

For D8-TNFα binding, protein A biosensors were pre-loaded at 4.5 nm with Infliximab at 10 μg/ml in TBS, equilibrated for 60 s, then loaded with purified StrepTagII-D8-TNFα at 100 nM for 5 min. Binding kinetic was then evaluated by exposing simultaneously biosensors to various concentrations (200, 100, 50, 25, 12.5, 6.25, 3.125 and 0 nM) of Histag-GFP or color variants.

Association and dissociation steps were measured for 5 min each. Unless otherwise specified, all steps were performed in TBS containing 0.002% Tween 20 and 0.01% BSA. Biosensors were regenerated using three cycles of alternating wash for 10 s in Glycine 10 mM pH 2.5 and in TBS. All the steps were run at 30°C with a continuous shake speed of 1000 RPM. The biosensor exposed to the 0 nM concentration was used as a background reference. Sensorgrams were processed using a single reference subtraction and analyzed using the Octet Data Analysis software 7.1 (ForteBio). Fitting was performed with a 1:1 binding fit model.

### Cell growth inhibition with TNFα recombinant proteins

Cell viability was measured using the XTT assay [39], based on a previously described assay for measuring TNFα activity [40]. Briefly, 2×10^4^ L929 cells were incubated for 4 h at 37°C and 5% CO_2_ in 96-well microtiter plates. TNFα recombinant proteins were diluted in Dulbecco's Modified Eagle's medium (DMEM) with 1% penicillin, 1% streptomycin, 10% heat-inactivated fetal bovine serum and 1 μg/ml actinomycin-D, and added to each well to give the final concentrations of 365 to 1.4 pM. After incubation for 20 h at 37°C and 5% CO_2_, 45 μL of 2,3-Bis-(2-Methoxy-4-Nitro-5-Sulfophenyl)-2*H*-Tetrazolium-5-Carboxanilide (XTT) labeling mixture (Roche Applied Science) were added to each well and the plates were incubated at 37°C for 4 h to allow color development. Optical density was recorded at 492 nm on a Varioskan reader (Thermo Scientific), using 690 nm as a reference wavelength.

## Results and Discussion

### Selection of anti-GFP specific binders

The Sac7d family, including homologous protein such as Sso7d, was proved highly flexible and tolerant as a host for the design of mutant libraries, allowing the generation of binders with high affinity and specificity against various targets from the randomization of either beta-sheet [16], or both non-extended [23] and extended [26] loops. These previous studies highlight the stability of this protein scaffold upon introduction of a large amount of mutations, including distinct library designs but also successful domain grafting between members of the OB-fold family members [24].

**Fig 1:**
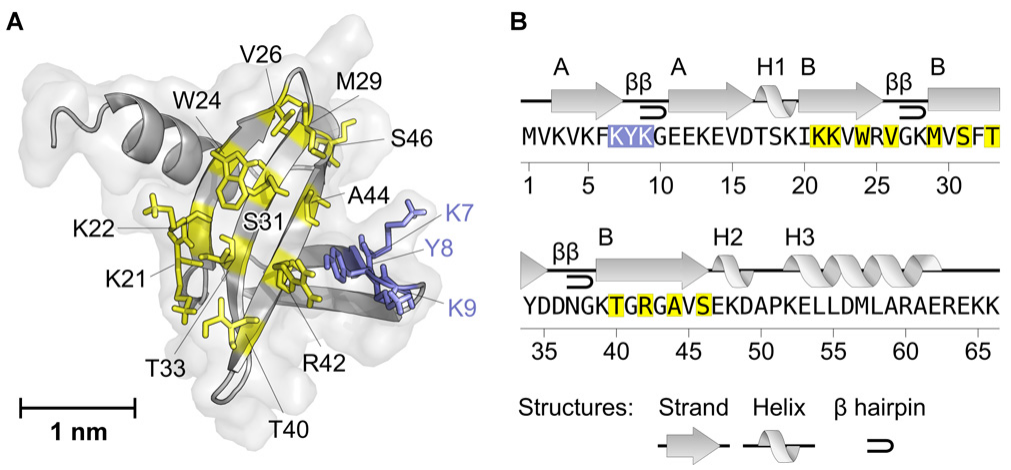
Structure of the Nanofitin OB-fold scaffold and randomized libraries. (A) Cartoon representation of wild-type Sac7d (Protein Data Bank code 1AZP). (B) Secondary structure plot of wild-type Sac7d. Helices are labeled H1, H2 and H3 and strands by their sheets A and B, from N-terminal to C-terminal extremities. Beta-turn motifs are indicated with β. Residues randomized in the library involved in the generation of anti-GFP Nanofitins are shown in (A) yellow sticks and (B) yellow frames. Residues of the first loop randomized in early library are shown in (A) purple sticks and (B) purple frames.

Early library design focused on the first loop and the second beta-sheet (Fig 1) of the Sac7d scaffold and consisted in the randomization of 14 residues [16], leading to a theoretical diversity of more than 1.6×10^18^ variants. Such diversity goes beyond the library size capacity of display technologies, generally considered to be of about 10^10^ for selection techniques involving transformed living cells (e.g. phage [41,42] or yeast display [43,44]) and 10^14^ for those *in vitro* such as ribosome display [45]. In order to increase the reproducibility of Sac7d variants selection by ribosome display, it seemed necessary to use a library that can be extensively explored. Reducing library size to 11 or less randomized positions, corresponding to 2×10^14^ variants, could fill this requirement. Such library size was actually sufficient enough to isolate OB-fold binders against various targets by yeast display, as previously described by Rao *et al*. with the randomization of 10 positions in the Sso7d scaffold [29,30,32]. Although the resulting binders present modest K_D_ (from 100 nM to μM), it is difficult to establish a direct correlation between the variations in affinity and the number of randomized positions since the scaffolds, targets and selection technique lightly differ.

Otherwise, the determination of Sac7d structure revealed that both N- and C-termini of the Nanofitin scaffold are available for conjugation as they are not involved in the binding site [46]. In this study, we made use of a Sac7d-based library consisting of 11 randomized positions (Fig 1) to generate a Nanofitin directed against GFP and its spectral variants (BFP, CFP and YFP), termed herein after GFP-ready tag, and provide a proof-of-concept of the use of Nanofitins as a fusion tag.

**Fig 2:**
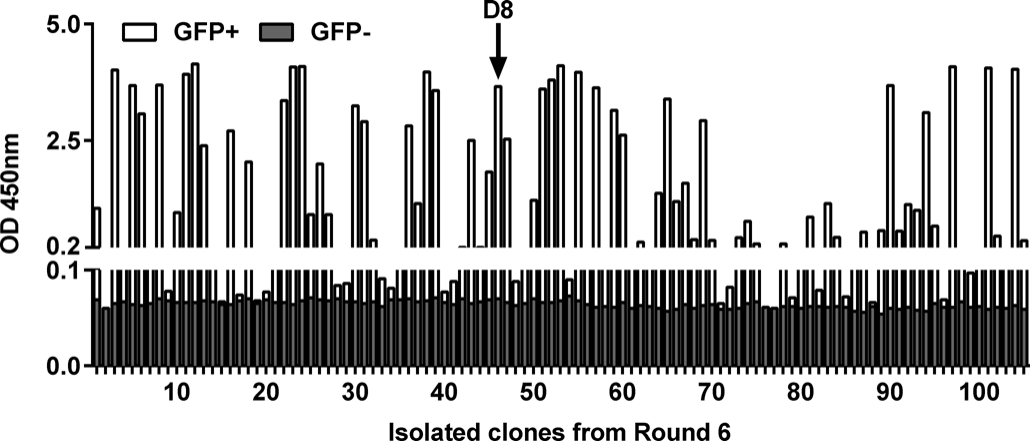
ELISA screen of the anti-GFP Nanofitins. Diversity from the sixth round of selection was screened by ELISA in presence (white bars) and absence of immobilized StrepTagII-GFP (superimposed grey bars). Anti-GFP Nanofitin D8 signals are indicated with an arrow.

After four rounds of selection by ribosome display, an ELISA screen was performed on isolated clones and 80/96 displayed a strong specific binding to the GFP (with at least a 10-fold signal increase upon GFP addition, S1 Fig), while poor sequence convergence was observed. To reduce the screening effort and narrow down the diversity to the Nanofitins with the best affinity, the selection was pushed for two additional rounds with decreasing concentration of the GFP protein bait. After six rounds, a second ELISA screening was completed on diluted crude culture extract of 105 isolated clones (Fig 2). More than 50 of the original 105 clones assayed were showing a specific response in presence of GFP with a signal of at least 10-fold the background measured in the absence of GFP. At this stage, all the Nanofitin hits were fused to an N-terminal Histag which allowed their ranking based on their expression yield by interferometry assay on octet RED96 with Ni-NTA sensors, using the same culture extracts. Considering the combination of ELISA response, expression yield as well as sequence enrichment, 6 hits were produced in flask cultures and purified to confirm their properties inferred from crude lysates. Eventually, focus was made on the Nanofitin D8 (highlighted with an arrow on Fig 2) for further characterizations and demonstration of the GFP-ready Nanofitin tag application, especially due to its high expression in bacterial culture (from 3 to 8-fold higher than other Nanofitins recovered after purification).

### Characterization of anti-GFP Nanofitin D8

#### Bacterial expression of soluble Nanofitin D8

Without optimization of the expression system, grams of anti-GFP Nanofitin D8 were purified from replicated flask cultures with an overall expression yield above 400 mg/l of culture, placing this clone beyond other anti-GFP hits and in the top expressed Nanofitins (average yield of Nanofitin expression in *E*. *coli* DH5α LacI_q_ is 40 mg/l of culture). Solubility of the purified Nanofitin D8 was also assessed by concentrating it to 169 mg/ml (18 mM) without observing precipitation or gelification. Such properties make D8 a potent candidate for applications at industrial scale. Otherwise, these results represent good hints regarding possible chimeric fusion of this Nanofitin to recombinant proteins, as the fusion would have a lower risk of altering expression and solubility in the expression host.

#### Autoclaving resistance

**Fig 3:**
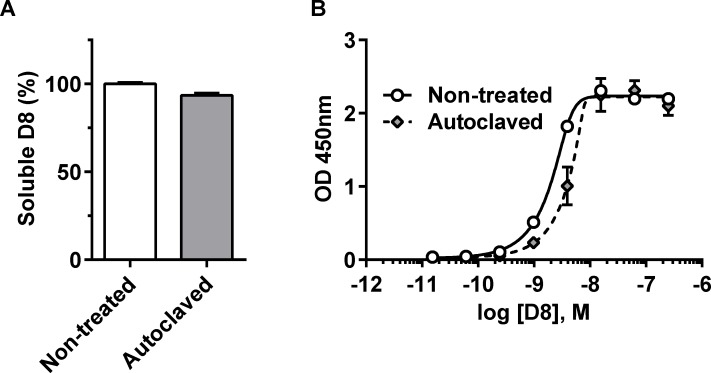
Anti-GFP Nanofitin D8 binding after heat-treatment by autoclaving. (A) Quantitation of soluble fraction of the Nanofitin D8 proteins before (white bar) and after (grey bar) autoclaving treatment (n = 3). (B) The interaction of purified Nanofitin D8 before and after sterilization process by autoclaving, respectively in plain and dashed lines, was assessed by ELISA with immobilized StrepTagII-GFP and concentration range of the Nanofitin (n = 3).

The characterization of anti-GFP Nanofitin D8 additionally focused on assessing its expected self-folding ability through its resistance to high temperatures exposure. From the promising features of Nanofitins, one of the most notable differences compared to other binding proteins (especially antibodies) is their resistance to extreme conditions of pH and temperatures. The natural environment of its original scaffold most likely explains this robustness. Nanofitins are derived from the Sac7d protein, originally discovered in *Sulfolobus acidocaldarius* archaebacteria [47]. In its host cytoplasm, wild-type Sac7d binds to DNA to protect it from melting, despite the surrounding 85°C conditions. Interestingly, this heat-resistance is conserved in Nanofitin variants, with average melting temperature above 80°C [16]. We evaluated residual activity of anti-GFP Nanofitin D8 after complete sterilization process by autoclaving to confirm its robustness. In spite of 121°C exposure for 20 min in solution, 93.5% of D8 remains soluble (Fig 3A) and is still fully active as determined by ELISA (Fig 3B).

Such results confirm the high resistance of Nanofitin binders, which could be a consequence of both its strong thermal resilience [16] and its renaturation ability [23,24]. Either explanation could rely on the self-folding capacity of the Sac7d scaffold that allows its full chemical synthesis [23,48]. In any case, the emanating properties of Nanofitins regarding their ease of folding suggest a strong compatibility with their use as a fusion partner.

#### Binding to fluorescent proteins from BFP to YFP

**Fig 4:**
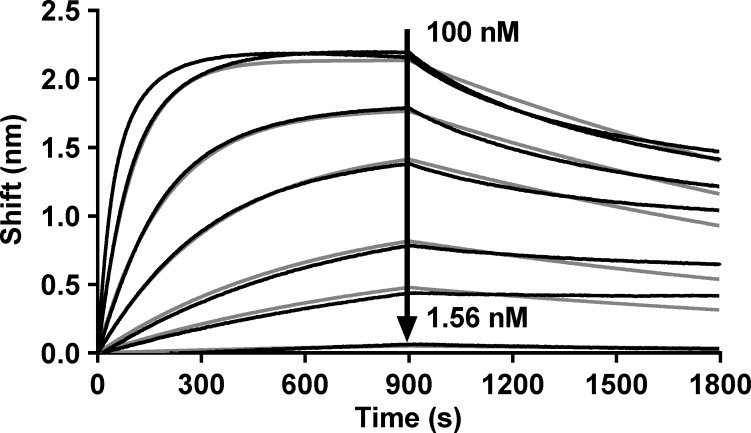
Affinity measurement of the Nanofitin D8 against StrepTagII-GFP. Kinetic characterization of StrepTagII-GFP–Anti-GFP Nanofitin D8 interaction, measured by interferometry. Black curves represent experimental data and grey curves represent the statistical fitting of the curves.

Besides its top rank based on expression yield and its thermal resistance, D8 was initially screened as one of the most affine anti-GFP Nanofitins. This affinity was more precisely determined with a biolayer interferometry assay using an immobilized Nanofitin and a concentration range of GFP (Fig 4). Model fitting to experimental data (R^2^ = 0.9957) resulted in equilibrium constant of dissociation (K_D_) equal to 2.52×10^−9^ M, given the following kinetic constants: k_on_ = 1.86×10^5^ M^-1^s^-1^ and k_off_ = 4.67×10^−4^ s^-1^.

Similar profiles were observed with spectral variants of enhanced GFP (S2 Fig). Anti-GFP Nanofitin D8 was tested and binds equally well to enhanced blue (EBFP), cyan (ECFP), green (EGFP) and yellow (EYFP) fluorescent proteins, with nM affinity range (Table 1).

**Table 1:** Kinetic constants of Nanofitin D8 directed toward GFP variants.

GFP variant	K_D_ (M)	k_on_ (M^-1^s^-1^)	k_off_ (s^-1^)	R^2^
GFP	2.52±0.01 ×10^−9^	1.86±0.01 ×10^5^	4.67±0.01×10^−4^	0.9957
EBFP	6.27±0.04 ×10^−9^	3.08±0.02 ×10^5^	1.93±0.01 ×10^−3^	0.9981
ECFP	5.83±0.04 ×10^−9^	2.63±0.01 ×10^5^	1.53±0.01 ×10^−3^	0.9988
EGFP	4.24±0.05 ×10^−9^	2.31±0.02 ×10^5^	9.79±0.08 ×10^−4^	0.9977
EYFP	4.46±0.04 ×10^−9^	2.70±0.01 ×10^5^	1.20±0.01 ×10^−3^	0.9984

Kinetic constants of interaction between Nanofitin D8 and GFP variants, determined by interferometry. k_on_, the association rate constant, in M^-1^s^-1^. k_off_, the dissociation rate constant in s^-1^. K_D_, the equilibrium binding constant, in M, computed as k_off_/k_on_. R^2^, the coefficient of determination of the fitted model.

### GFP-ready tagged human TNFα

#### Expression and purification of soluble TNFα fusion

**Fig 5:**
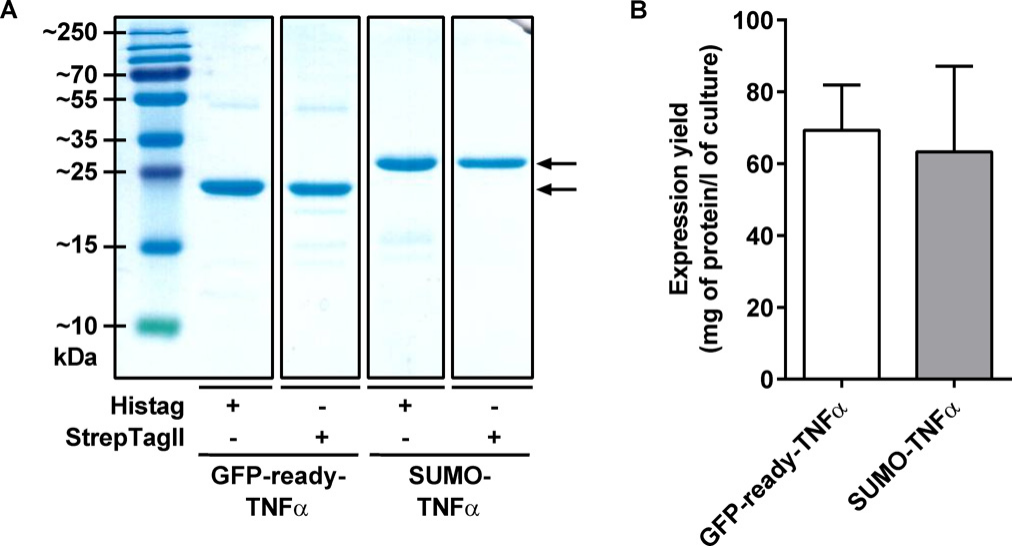
Expression of soluble TNFα recombinant proteins in *E*. *coli*. (A) SDS-PAGE profiles with 2 μg of purified TNFα fusions, after Coomassie blue staining. Bands indicated with an arrow correspond to the expected recombinant proteins. From left to right: Histag GFP-ready-TNFα (MW: 26.5 kDa), StrepTagII GFP-ready-TNFα (MW: 26.6 kDa), Histag SUMO-TNFα (MW: 30.0 kDa), StrepTagII SUMO-TNFα (MW: 30.1 kDa). (B) Expression yields of Histagged GFP-ready-TNFα and SUMO-TNFα. Quantitation performed by biolayer interferometry with Ni-NTA sensors (n = 4).

In order to demonstrate the possible use of a Nanofitin as a fusion tag, GFP-ready tagged TNFα (human tumor necrosis factor alpha) was compared to the SUMO (Small Ubiquitin-like Modifier protein) tagged TNFα [38] and recombinant untagged TNFα from a commercial source. We assessed the integrity of the carrier TNFα protein as well as the ability of the GFP-ready tag to maintain its binding properties for the different GFP variants when fused to a partner. GFP-ready-TNFα and SUMO-TNFα were expressed in *E*. *coli* in flask and purified to high homogeneity according to the purification tag fused to their N-terminal extremity (Fig 5A). Interestingly, a similar expression yield of more than 60 mg/l of culture was observed regardless of the fusion tag (GFP-ready or SUMO), as determined with interferometry assay (Fig 5B).

#### Binding to fluorescent proteins and TNFα ligand

**Fig 6:**
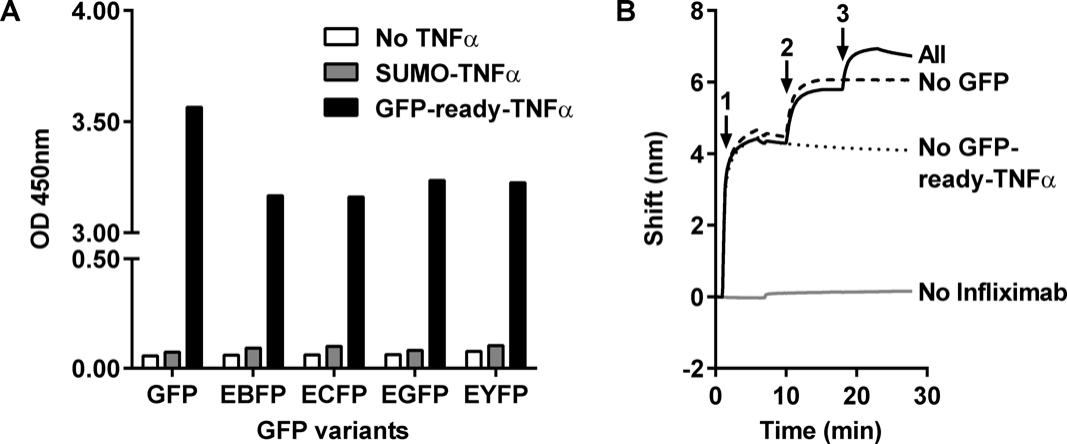
Binding of TNFα fusions to anti-TNFα antibody and GFP variants. Binding of GFP variants to TNFα fusions, measured by ELISA or interferometry with immobilized anti-TNFα antibody, Infliximab. (A) ELISA with no TNFα fusion (white bars), SUMO-TNFα fusion (grey bars) or GFP-ready-TNFα fusion (black bars) captured in plate wells (n = 3). (B) Interferometry kinetic binding profile with loading of Infliximab (step 1), GFP-ready-TNFα fusion (step 2) and GFP (step 3). Besides the sample with all bound partners (plain line), controls without GFP, GFP-ready-TNFα or Infliximab were also measured (dashed line, dotted line and grey line, respectively).

Non-IgG anti-GFP binders have been found really useful for the characterization of protein-protein interactions with regard to the natural complexity of living cells compartments, as highlighted with the Nanobody-based fluorescent-three-hybrid strategy [9]. Such application partly relies on the simultaneous binding of an anti-GFP binder and a functional carrier protein, respectively with the GFP and a natural partner. To characterize the GFP-ready tag within the scope of a similar use of anti-GFP binders, we modeled such multilayer interaction pattern by ELISA and interferometry sandwich assays that involve the concomitant binding of anti-GFP and TNFα moieties. Therapeutic anti-TNFα monoclonal antibody (Infliximab) was immobilized to capture GFP-ready-TNFα recombinant proteins and the binding of GFP and spectral variants was measured. SUMO-TNFα construct was used as a control in the ELISA system to highlight that the GFP-ready tag provided the specific binding to the GFP spectral variants. While no ELISA signal (Fig 6A) was observed with the immobilization of SUMO-TNFα or in absence of TNFα (grey and white bars, respectively), immobilization of GFP-ready-TNFα (black bars) allowed the capture of the different GFP variants, providing a saturating ELISA signal in each case.

To further characterize the GFP-ready-TNFα construct, the setup was reproduced and analyzed by biolayer interferometry with additional controls (Fig 6B). The lack of the capture antibody Infliximab completely abolished the system showing, firstly, the absence of non-specific binding to the surface by the subsequent components and, secondly, the involvement of Infliximab in the immobilization of the TNFα construct. At the same time, this efficient capture brings first evidence in the preservation of TNFα integrity upon fusion to the GFP-ready tag. GFP (Fig 6B) and its spectral variants (S3 Fig) were bound to the surface only in the presence of the GFP-ready-TNFα, which further supports the previous ELISA results showing that the anti-GFP Nanofitin D8 kept its binding capacity when used as a GFP-ready tag and fused to another molecular partner. For a more quantitative investigation, this interferometry assay setup was also performed for the affinity calculation of the GFP-ready-TNFα for the GFP variants (Table 2). Affinities of the GFP-ready-TNFα for different GFP variants were found similar to the one previously determined with the Nanofitin D8 alone, demonstrating this anti-GFP Nanofitin is able to efficiently pass down its full binding capacity to the recombinant protein fused with to the GFP-ready tag.

**Table 2:** Kinetic constants of GFP-ready-TNFα directed toward GFP variants.

GFP variant	K_D_ (M)	k_on_ (M^-1^s^-1^)	k_off_ (s^-1^)	R^2^
GFP	2.62±0.01 ×10^−9^	2.23±0.01 ×10^5^	5.85±0.02 ×10^−4^	0.9997
EBFP	6.75±0.03 ×10^−9^	2.78±0.01 ×10^5^	1.88±0.01 ×10^−3^	0.9981
ECFP	5.93±0.03 ×10^−9^	2.59±0.01 ×10^5^	1.54±0.01 ×10^−3^	0.9986
EGFP	5.35±0.02 ×10^−9^	1.66±0.01 ×10^5^	8.87±0.03 ×10^−4^	0.9994
EYFP	4.76±0.02 ×10^−9^	2.18±0.01 ×10^5^	1.04±0.00 ×10^−3^	0.9990

Kinetic constants of interaction between GFP-ready-TNFα and GFP variants, determined by interferometry. k_on_, the association rate constant, in M^-1^s^-1^. k_off_, the dissociation rate constant in s^-1^. K_D_, the equilibrium binding constant, in M, computed as k_off_/k_on_. R^2^, the coefficient of determination of the fitted model.

We demonstrated *in vitro* that the GFP-ready tag fully retains the binding capacity of the Nanofitin D8 towards the GFP variants when fused to a carrier protein, while not interfering with the interaction between the carrier protein and a natural partner. From this demonstration and the inherent properties of Nanofitins, we expect the GFP-ready tag to be a suitable tool for *in vitro* and live cell applications, in addition to other available artificially derived anti-GFP binders [6–11,35]. We focused on the Nanofitin D8 for this proof-of-concept, but other anti-GFP Nanofitins are available and the Nanofitin chosen for the GFP-ready tag could depend on the specific application and/or the fusion protein. During the selection process, we identified a pair of anti-GFP Nanofitins that do not overlap in respect to their binding site on the GFP (data not shown), which open up possibilities of using the GFP-ready tag technology for the reconstitution of functional activity *in cellulo*, as demonstrated with Nanobodies by Tang *et al*. [49]. Furthermore, we assume that the GFP-ready tag can be a valuable tool as a substitute to the direct fusion with GFP. This alternative approach should bypass some of the inherent issues of GFP fusions, especially regarding their expression and aggregation [50] by replacing the GFP with the more stable Nanofitin moiety. Moreover, the indirect labeling provided by the binding to chosen GFP variants should allow the customization and renewal of the source of fluorescence outside of the cellular environment, particularly convenient to circumvent photobleaching issues of the chimeric proteins [51]. Interestingly, the GFP-ready system also divides the size of the fusion tag by 4 as compared to GFP fusion, what we anticipated results in the narrowing down of steric effects on its fused partner prior to GFP indirect labeling. Such GFP-ready tag tool could be beneficial in experimental setups with recombinant membrane proteins, allowing to monitor their presence on the cell surface with a customizable range of excitation and emission spectra (BFP, CFP, GFP or YFP) from a unique and simple fusion, while proving a lesser risk to hinder the ability of the carrier protein to interact with its potential ligands in absence of the GFP variants.

#### Impact on TNFα activity

**Fig 7:**
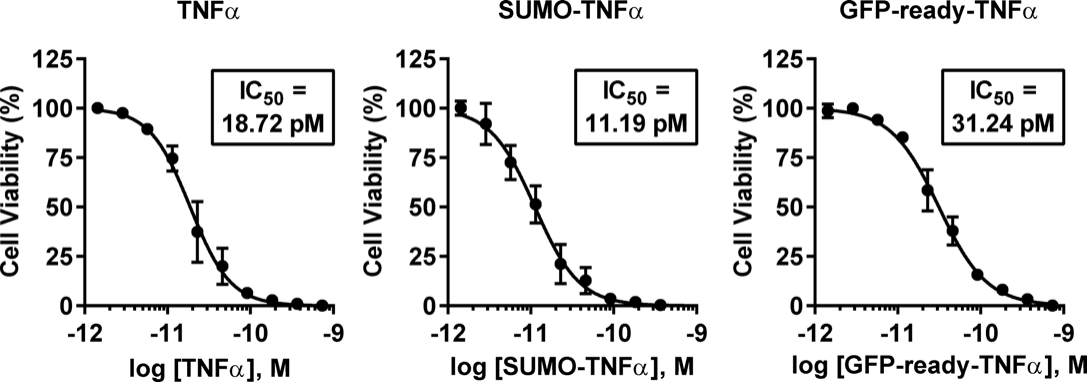
Cell growth inhibition of TNFα fusions. TNFα activity was assessed by measuring cell viability (n = 3) using the XTT assay on L929 sensitized cells with actinomycin-D and concentration range of TNFα fusions (untagged soluble TNFα, SUMO-TNFα or GFP-ready-TNFα from left to right). Individual IC_50_ values were determined from curve fitting.

Integrity of the TNFα moiety in the GFP-ready-TNFα construct was further investigated by directly measuring its activity in a cell growth inhibition assay on L929 cells (Fig 7). In presence of actinomycin-D, TNFα promotes the apoptosis induction of the TNFR1-expressing L929 cells. Activity of the GFP-ready-TNFα (right panel) was compared to SUMO-TNFα (center panel) and untagged TNFα (left panel) from commercial source over a range of concentrations from 365 to 1.4 pM. All the constructs were found to be active regardless of the presence of a fusion partner, with half maximal inhibitory concentrations (IC_50_) values of a similar order of magnitude, ranging from 11.19 to 31.24 pM.

The functional assay with GFP-ready-TNFα strengthened the validation of simultaneous binding to both GFP-ready tag and TNFα ligands. Thus, it demonstrates a specific addition of a new feature without affecting original activity of a recombinant protein. Obviously, GFP-ready tag fusion will be subject to the same parameters that rule chimeric constructs to be applicable with other recombinant proteins. The broad use of this technology may need some optimization on a case-by-case basis regarding fusion components, including the choice of fused termini and the linker composition or length. However, this study proved promising results and, as a matter of fact, represents the first successful demonstration of the Nanofitin-based GFP-ready tag.

## Conclusions

We described the fully *in vitro* generation of an anti-GFP Nanofitin, called GFP-ready tag, with nanomolar affinity for green fluorescent protein (GFP) but also for its blue (BFP), cyan (CFP), or yellow (YFP) spectral variants. Usefulness of non-IgG anti-GFP binders as a research tool is not anymore to be demonstrated, and mostly relies on their capability to serve as a fusion partner expressing and folding in the different compartments of a cell. While most of the studies on anti-GFP scaffolds are application-oriented, we rather focused on the characterization of the GFP-ready tag as a generic fusion technology by using TNFα as a model carrier protein. Besides its efficient binding, this anti-GFP shows the inputs provided by the Nanofitin alternative scaffold, such as a small cysteine-free single chain structure and a remarkable resistance profile, demonstrated by its full resilience upon a cycle of thermal sterilization process. These results suggest a robust structure and/or a proper self-folding capacity of the Nanofitin that allow the GFP-ready tag to be used in multiple contexts, including its production as chimeric fusion in either prokaryotic or eukaryotic system, but also by full chemical synthesis.

In this proof-of-concept study, we demonstrated that an anti-GFP Nanofitin can be expressed in fusion with human TNFα, and both the Nanofitin and the carrier protein remain fully active in the chimeric construct. The GFP-ready tag has been shown equivalent to the well-known SUMO tag in regard to the recovery of soluble chimeric construct and the preservation of the activity. We acknowledge that the fusion-friendly features of Nanofitins were demonstrated within the limits of the model framework that has been used here. Therefore, their applicability remains to be defined on a case-by-case basis for other carrier proteins, emphasizing the interest of having different anti-GFP scaffolds available. While Nanobodies are less likely to carry a fusion partner on their N-terminal extremity due to its close proximity with their binding site, both extremities of Nanofitins are readily available for the genetic fusion to a carrier. The resulting flexibility for designing chimeric fusions might further extend the scope of protein partners that could benefit from the anti-GFP technology, especially in cell biology application. Researchers would be able to explore multiple scaffolds and designs, which are often a criterion of success in the construction of a chimeric fusion, as there is no universal solution.

Finally, the flexibility and robustness of Nanofitins illustrate a wider concept of Nanofitin-based functionalization, which could be extended to other fused molecular partners and/or Nanofitins of other specificities.

## Supporting Information

S1 Appendix Construction of histagged human Tumor-Necrosis Factor alpha fusions. Materials and methods appendix for the construction of Histag SUMO-TNFα and Histag GFP-ready-TNFα expression vectors.
(DOCX)

S1 Fig ELISA screen of the anti-GFP Nanofitins after fourth round of selection. Diversity from the fourth round of selection was screened by ELISA with undiluted lysates in presence (white bars) and absence of immobilized StrepTagII-GFP (superimposed grey bars).
(TIF)

S2 Fig Spectral variants of enhanced GFP. Comparison of spectral variants of enhanced GFP: Green (EGFP), Blue (EBFP), Cyan (ECFP) and Yellow (EYFP) variants of enhanced GFP. (A) Sequence alignment, with mutations highlighted in colored frames. (B) Normalized spectra of fluorescence excitation. (C) Normalized spectra of fluorescence emission.
(TIF)

S3 Fig Kinetic binding profiles of TNFα fusions to anti-TNFα antibody and enhanced fluorescent protein variants. Interferometry kinetic binding profile with loading of Infliximab (step 1), GFP-ready-TNFα fusion (step 2) and enhanced fluorescent protein variants (step 3). Besides the sample with all bound partners (plain line), controls without enhanced fluorescent protein variant, GFP-ready-TNFα or Infliximab were also measured (dashed line, dotted line and grey line, respectively). Binding with EBFP, ECFP, EGFP and EYFP variants are shown in panel (A), (B), (C) and (D), respectively.
(TIF)
