# Supplementary material for: Use of the Nanofitin Alternative Scaffold as a GFP-Ready Fusion Tag
Source: PLoS One. 2015 Nov 5;10(11):e0142304. doi: 10.1371/journal.pone.0142304 (PMC4634965; doi:10.1371/journal.pone.0142304)
Supplement: S1 Appendix — Materials and methods appendix for the construction of Histag SUMO-TNFα and Histag GFP-ready-TNFα expression vectors. (DOCX) [file pone.0142304.s001.docx]

Use of the Nanofitin alternative scaffold as a GFP-ready fusion tag

*Simon Huet^1^, Harmony Gorre^1^, Anaëlle Perrocheau^1^, Justine Picot^1^, Mathieu Cinier^1*^*

^1^Affilogic SAS, Nantes, France

* Corresponding author

Email: mathieu@affilogic.com

# Supporting Information

## Appendix S1. Construction of histagged human Tumour-Necrosis Factor alpha fusions

Gene coding for the chimeric construct SUMO-TNFα was sub-cloned in pQE30 vector by Gibson assembly, resulting in a Histag-SUMO-TNFα construct. The vector and the TNFα coding sequence were amplified by PCR using, respectively, the pairs of oligonucleotides Gpls01C_For (TAATGACTGAGCTTGGACTCC) and Gpls01N_Rev (GTGATGCGATCCT CTCATAG), or NHis_SUMO_For (GAGAGGATCGCATCACCATCACCATCACGGATC CATGTCGGACTCAGAAGTCAATCAAG) and TNF_Stop_Rev (GAGTCCAAGCTCAGT CATTACAGCGCAATAATGCCAAAATAC). Linearized vector (100 ng) was mixed with 3 molar equivalents of the gene insert in a final volume of 5 µl. Then, 15 µL of the Gibson assembly mix (25% PEG-8000, 500 mM Tris-HCl, 50 mM MgCl2, 50 mM DTT, 1 mM Mix dNTPs, 5 mM NAD, 2U of T5 exonuclease, 12.5U of Phusion polymerase, 2000U of Taq ligase) were added and the solution was incubated for 1 hour at 50 °C. *E. coli* DH5α LacIq strains (Invitrogen) were transformed with 10 µl of the resulting material. Clones were selected on 2xYT medium plates containing 100 µg/ml ampicillin and 25 µg/ml kanamycin.

Construction of Histag-GFP-ready-TNFα followed a similar procedure but the TNFα coding sequence was sub-cloned in pQE30 vector containing Histag-D8 coding sequence (obtained after isolation of clones of the sixth selection round, as described in “Materials and Methods”). The vector and the TNFα coding sequence were amplified by PCR using, respectively, the pairs of oligonucleotides Gpls01C_For and D8_GS_Rev (GCTACGCACCGAGCCCTTTTTCTCGCGTTCCGCA), or GS_TNF_For (GGCTCGGTG CGTAGCAGCAGCC) and TNF_Stop_Rev.

All the constructions were confirmed by Sanger sequencing (GATC biotech).
