# Supplementary figures and images for: Use of the Nanofitin Alternative Scaffold as a GFP-Ready Fusion Tag
Source: PLoS One. 2015 Nov 5;10(11):e0142304. doi: 10.1371/journal.pone.0142304 (PMC4634965; doi:10.1371/journal.pone.0142304)

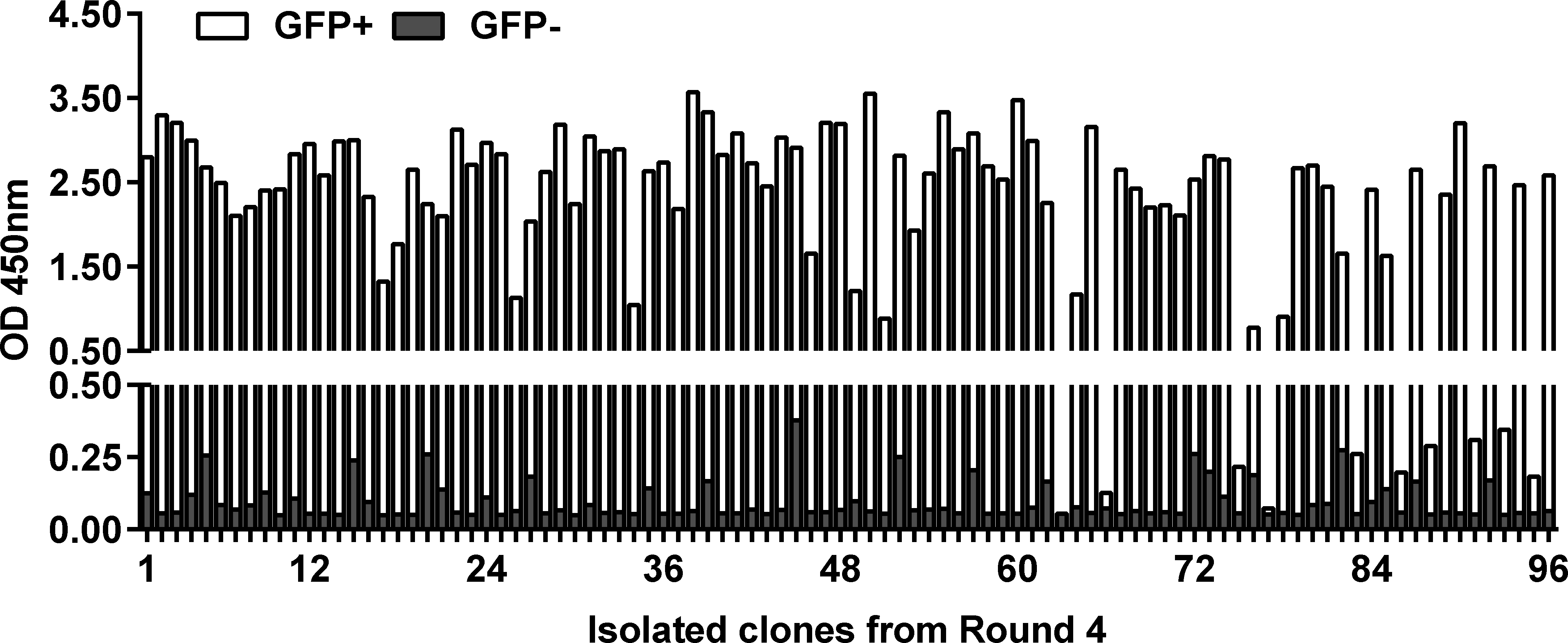

Supplement: S1 Fig — Diversity from the fourth round of selection was screened by ELISA with undiluted lysates in presence (white bars) and absence of immobilized StrepTagII-GFP (superimposed grey bars). (TIF) [file pone.0142304.s002.tif]

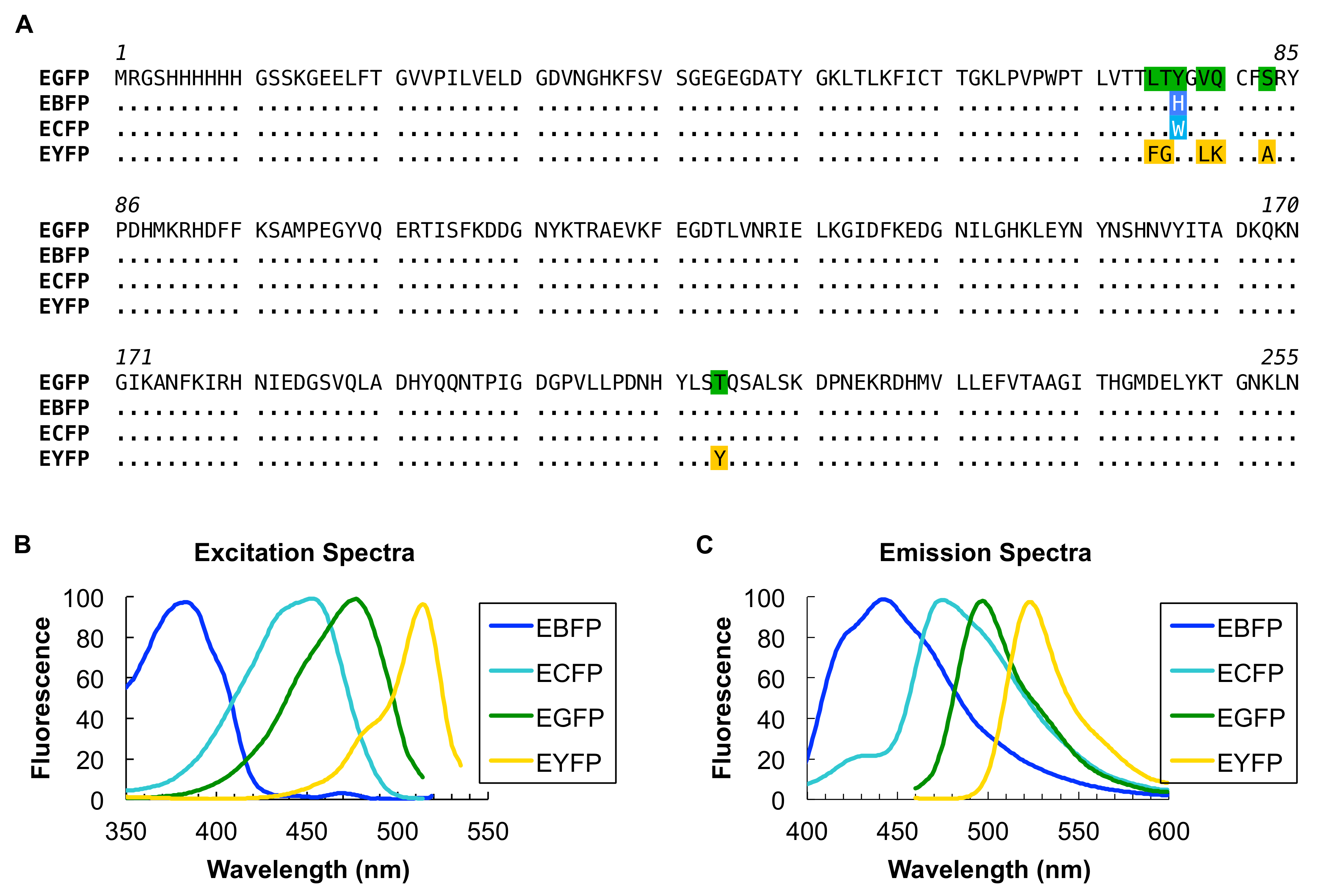

Supplement: S2 Fig — Comparison of spectral variants of enhanced GFP: Green (EGFP), Blue (EBFP), Cyan (ECFP) and Yellow (EYFP) variants of enhanced GFP. (A) Sequence alignment, with mutations highlighted in colored frames. (B) Normalized spectra of fluorescence excitation. (C) Normalized spectra of fluorescence emission. (TIF) [file pone.0142304.s003.tif]

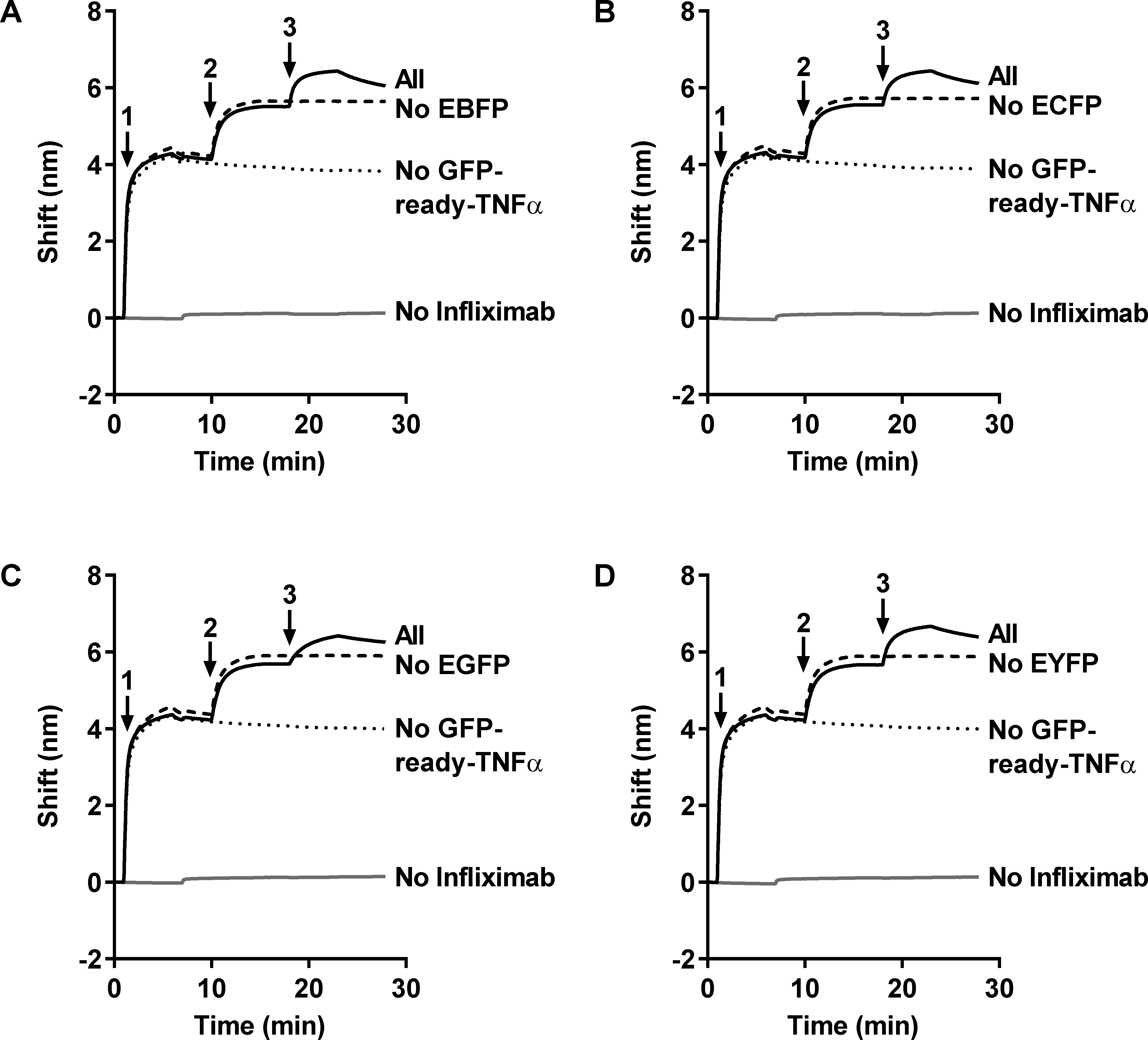

Supplement: S3 Fig — Interferometry kinetic binding profile with loading of Infliximab (step 1), GFP-ready-TNFα fusion (step 2) and enhanced fluorescent protein variants (step 3). Besides the sample with all bound partners (plain line), controls without enhanced fluorescent protein variant, GFP-ready-TNFα or Infliximab were also measured (dashed line, dotted line and grey line, respectively). Binding with EBFP, ECFP, EGFP and EYFP variants are shown in panel (A), (B), (C) and (D), respectively. (TIF) [file pone.0142304.s004.tif]
